# Treatment options for venous thromboembolism: lessons learnt from clinical trials

**DOI:** 10.1186/s12959-014-0027-8

**Published:** 2014-12-08

**Authors:** Simon McRae

**Affiliations:** Department of Haematology, SA Pathology, Royal Adelaide and Queen Elizabeth Hospitals, Frome Rd, Adelaide, SA 5000 Australia

**Keywords:** Apixaban, Dabigatran, Disease management, Edoxaban, Rivaroxaban, Venous thromboembolism

## Abstract

Venous thromboembolism (VTE), comprising deep vein thrombosis and pulmonary embolism, is a common condition associated with a significant clinical and economic burden. Anticoagulant therapy is the mainstay of treatment for VTE, having been shown to reduce the risk of death in patients with pulmonary embolism, and recurrence or extension of thrombi in patients with deep vein thrombosis during the initial treatment period. Long-term anticoagulation is indicated in some individuals with VTE, depending on individual risk of VTE recurrence and anticoagulant-related bleeding. Management of VTE in clinical practice is often complex because patients’ characteristics and treatment needs may differ considerably from those encountered in clinical trials. Current guidelines recommend the use of either low molecular weight heparins or fondaparinux overlapping with and followed by a vitamin K antagonist for the initial treatment of VTE, with the vitamin K antagonist continued when long-term anticoagulation is required. These traditional anticoagulants have practical limitations that have led to the development of direct oral anticoagulants that directly target either Factor Xa or thrombin and are administered at a fixed dose without the need for routine coagulation monitoring. This review discusses practical considerations for hospital physicians and haematologists in the management of VTE treatment, including the potential for the direct oral anticoagulants to simplify treatment.

## Introduction

Venous thromboembolism (VTE), comprising deep vein thrombosis (DVT) and pulmonary embolism (PE), is the third most common cardiovascular disease after myocardial infarction and stroke [1], and hence a significant cause of morbidity and mortality worldwide. The estimated annual incidence of VTE is one to two cases per 1000 in the general population, although annual incidences as high as four per 1000 have been reported [2,3]. Potentially, the true incidence of VTE is under-reported, because of the failure to identify silent PE *ante mortem* and the falling rate of autopsy [2,4-6]. VTE is associated with a significant economic burden owing to its prevalence, cost of treatment, and potential for recurrence and long-term complications. VTE-related healthcare costs in the US alone have been variously estimated at between $2 billion and $10 billion per year [2,3,7,8].

VTE occurs in men and women of all ethnicities and ages, although incidence rates vary between groups and are considerably higher among the elderly [5,9]. The incidence, and hence associated cost, of VTE is likely to increase in many societies because of an ageing population [3]. In the US, the first incidence of VTE was shown to rise exponentially from <5 cases per 100,000 persons <15 years of age to ~500 cases (0.5%) per 100,000 persons at age 80 years [5], and studies across Asia and Europe demonstrate a similar trend [10-12].

Known risk factors for VTE are listed in Table 1. Identification of at-risk individuals and use of appropriate thromboprophylactic measures has been shown to reduce the incidence of VTE [4,13]; however, use of thromboprophylaxis remains suboptimal [13-15]. Furthermore, 25–50% of VTE cases occur in the absence of an identifiable risk factor and are considered unprovoked [2]. Therefore, even with optimal use of thromboprophylaxis, the community burden of VTE will remain significant, and optimal treatment to minimize morbidity and mortality associated with the condition will remain important.

**Table 1:** **Risk factors for venous thromboembolism** [2,16-19]

**Genetic**	
Factor V Leiden	Prothrombin G20210A
Antithrombin deficiency	Prothrombin C deficiency
Family history of venous thromboembolism	Prothrombin S deficiency
Hyperhomocysteinaemia	Sickle cell trait
**Acquired, non-transient**	
Increased age	Spinal cord injury
Obesity	Prior venous thromboembolism
Chronic medical illness	Central venous lines
Cancer	Transvenous pacemaker
Antiphospholipid antibodies	Neurological disease with leg paresis
Heparin-induced thrombocytopenia	
Myeloproliferative disorders	
**Acquired, transient**	
Surgery	Medications, including
Trauma	hormonal contraceptives,
Fractures	hormone therapy,
Immobilization	chemotherapy drugs,
Pregnancy and childbirth	epoietin-alpha and
Red blood cell transfusion	darbepoietin-alpha

This article provides an overview of the practical considerations involved in initial and long-term management of patients diagnosed with VTE. It will also focus on the potential impact of direct oral anticoagulants on the management of VTE.

### Initial management of venous thromboembolism

It has been suggested recently that treatment of VTE can be divided into two phases: an initial “active treatment” phase of 3 months and a subsequent “secondary prevention phase” [20]. Anticoagulation during the initial treatment period has been shown to reduce the risk of initial or additional embolization in patients with proximal DVT or PE, and to reduce the risk of death in patients with PE [21,22]. Suboptimal anticoagulation during this initial 3-month period has been associated with an increased risk of recurrent thrombosis [23], with the risk highest if anticoagulation is inadequate during the first month of treatment [24]. Therefore, anticoagulation is the mainstay of the initial treatment of VTE.

The American College of Chest Physicians (ACCP) guidelines, the most widely accepted recommendations on VTE treatment, recommend that patients with acute proximal DVT or PE receive an initial standard anticoagulant regimen consisting of the administration of a parenteral anticoagulant (subcutaneous [s.c.] low molecular weight heparin [LMWH] or fondaparinux, or intravenous [i.v.] or s.c. unfractionated heparin [UFH]) for at least 5 days, with early initiation of a vitamin K antagonist (VKA) such as warfarin [25]. According to European guidelines, parenteral anticoagulation should be continued until an international normalized ratio (INR) of at least 2.0 has been achieved on 2 consecutive days, because of the slow onset of action of the antithrombotic effect of VKAs [26]. Although there is evidence that initial use of LMWH is associated with a lower risk of recurrent thrombosis, major bleeding and mortality compared with i.v. UFH [27], the recommendation and widespread adoption of LMWHs as the agent of choice has also been driven by the ability to administer them at a fixed weight-based s.c. dose, thereby allowing outpatient therapy in low-risk patients.

In patients with proximal DVT or PE, anticoagulant treatment is recommended to be continued for at least 3 months [25]. A shorter duration of therapeutic anticoagulation than this is associated with an increase in the risk of recurrent thrombosis, which in turn increases the risk of the chronic complications of post-thrombotic syndrome (PTS) and chronic thromboembolic pulmonary hypertension. In patients with provoked isolated distal DVT, anticoagulation can probably be stopped after a shorter duration of 4–6 weeks without increasing the risk of recurrent events [25]. The decision to prolong anticoagulation past this initial 3-month treatment period is determined by the balance between the risk of bleeding with ongoing anticoagulation and the risk of recurrent thrombosis in patients who stop taking anticoagulant therapy. Factors influencing this decision and alternative long-term anticoagulant options will be discussed in a subsequent section.

Although the above treatment strategy of an initial parenteral anticoagulant overlapped with and followed by a VKA for a minimum of 3 months is generally safe and effective, with a rate of recurrent VTE of 3–4% and a major bleeding rate of ~1% [27], this approach has known limitations. The requirement for the initial use of a parenteral anticoagulant has practical implications, and requires additional resource utilization when a patient cannot self-inject, but is otherwise suitable for home therapy. Because of the narrow therapeutic window and variable dose response, influenced by both genetic and environmental factors [28,29], warfarin and other VKAs require regular coagulation monitoring and dose adjustment to maintain an INR within the therapeutic range (INR 2.0–3.0), complicating their use in day-to-day care [30]. It has been suggested that patient self-testing and self-management of VKA therapy has the potential to improve clinical outcomes through improvements in time spent in therapeutic range and reduce healthcare resource utilization. In a systematic review of studies enrolling patients receiving warfarin for a number of indications, self-testing using a point-of-care INR measurement device and self-dose adjustment using a pre-set algorithm significantly reduced the rate of major thrombotic complications compared with standard laboratory-based monitoring, with no significant difference in the rate of major bleeding or all-cause mortality. Self-testing alone did not result in a significant change in thromboembolic outcomes and did not have such a high impact on cost savings [31]. Nonetheless, not all patients are suitable for self-management of VKA therapy (such as the very elderly), and selected patients must be highly motivated and trained appropriately [31].

### Direct oral anticoagulants for the initial treatment of venous thromboembolism

The above practical limitations of traditional anticoagulants have helped to drive the development and evaluation in large phase III trials of direct oral anticoagulants, which have a more predictable anticoagulant effect allowing for fixed-dose administration without the need for routine coagulation monitoring or dose adjustment. These agents directly target specific steps in the coagulation cascade and inhibit either Factor Xa (rivaroxaban, apixaban and edoxaban) or thrombin (dabigatran). All have been evaluated for the initial treatment or long-term prevention of VTE in phase III studies. Rivaroxaban, dabigatran and apixaban have been approved for the treatment of DVT and PE, and the prevention of recurrent DVT and PE, in adults in the European Union and the US. Edoxaban is approved for this indication in Japan, and it is reasonable to assume that it will receive wider approval for VTE treatment in the future (Table 2).

**Table 2:** **Phase III trials of direct oral anticoagulants in the treatment of venous thromboembolism** [32-38]

**Drug**	**Indication**	**Study name**	**Treatments**	**Main findings**
Rivaroxaban	Acute treatment of DVT without PE	EINSTEIN DVT [32]	Rivaroxaban 15 mg bid for 3 weeks, then 20 mg od for 3–12 months or Enoxaparin for ≥5 days with VKA (target INR 2.0–3.0) for 3–12 months	VTE recurrence for rivaroxaban vs LMWH/VKA 2.1% vs 3.0% (HR 0.68; 95% CI 0.44–1.04; p < 0.001 for non-inferiority; p = 0.08 for superiority). Major or non-major clinically relevant bleeding for rivaroxaban vs LMWH/VKA 8.1% vs 8.1% (HR 0.97; 95% CI 0.76–1.22; p = NS)
	Acute treatment of PE with or without DVT	EINSTEIN PE [33]	Rivaroxaban 15 mg bid for 3 weeks, then 20 mg od for 3–12 months or Enoxaparin for ≥5 days with VKA (target INR 2.0–3.0) for 3–12 months	VTE recurrence for rivaroxaban vs LMWH/VKA 2.1% vs 1.8% (HR 1.12; 95% CI 0.75–1.68; p = 0.003 for non-inferiority). Major or non-major clinically relevant bleeding for rivaroxaban vs LMWH/VKA 10.3% vs 11.4%; p = NS
	Secondary prevention of VTE	EINSTEIN EXT [32]	After completion of 6–12 months’ treatment: Rivaroxaban 20 mg od for 6 or 12 months or Placebo for 6 or 12 months	VTE recurrence for rivaroxaban vs placebo 1.3% vs 7.1% (HR 0.18; 95% CI 0.09–0.39; p < 0.001 for superiority). Major bleeding for rivaroxaban vs placebo 0.7% vs 0% (HR not available; p = 0.11)
Apixaban	Acute treatment of VTE	AMPLIFY	Apixaban 10 mg bid for 7 days then 5 mg bid for 6 months or Enoxaparin 1 mg/kg (s.c.) bid until INR ≥2, warfarin (target INR 2.0–3.0)	VTE recurrence or VTE-related death for apixaban vs LMWH/warfarin 2.3% vs 2.7% (RR 0.84; 95% CI 0.60–1.18; p < 0.001 for non-inferiority for apixaban). Major bleeding for apixaban vs LMWH/warfarin 0.6% vs 1.8% (RR 0.31; 95% CI 0.17–0.55; p < 0.001 for superiority for apixaban)
	Secondary prevention of VTE	AMPLIFY-EXT [37]	After 6–12 months of apixaban or warfarin: Apixaban 2.5 mg or 5 mg bid for 12 months or Placebo for 12 months	VTE recurrence or any-cause death for apixaban 2.5 mg or 5.0 mg vs placebo, 3.8% or 4.2% vs 11.6% (2.5 mg RR 0.33; 95% CI 0.22–0.48; 5 mg RR 0.36; 95% CI 0.25–0.53; p < 0.001 for superiority for both apixaban doses). Major bleeding for apixaban 2.5 mg or 5.0 mg vs placebo, 0.2% (RR 0.49; 95% CI 0.09–2.64) or 0.1% (RR 0.25; 95% CI 0.03–2.24) vs 0.5% (p values not available)
Edoxaban	Acute treatment of VTE	Hokusai-VTE [39]	LMWH or UFH for ≥5 days then edoxaban 60 mg od for 3–12 months or LMWH or UFH for ≥5 days then warfarin (target INR 2.0–3.0) for 3–12 months	VTE recurrence or VTE-related death for edoxaban vs warfarin 3.2% vs 3.5% (HR 0.89; 95% CI 0.70–1.13; p < 0.001 for non-inferiority for edoxaban). First major or non-major clinically relevant bleeding event 8.5% vs 10.3% (HR 0.81; 95% CI 0.71–0.94; p = 0.004 for superiority for edoxaban)
	Acute treatment of VTE	eTRIS (NCT01662908)	Edoxaban 90 mg od for 10 days then 60 mg od (total 90 days) or Warfarin (target INR 2.0–3.0) for 90 days, with enoxaparin or UFH for ≥5 days until target INR reached	Ongoing
Dabigatran	Acute treatment of VTE	RE-COVER [35]	LMWH or UFH for ≥5 days; dabigatran 150 mg bid for 6 months or LMWH or UFH for ≥5 days; warfarin (target INR 2.0–3.0) for 6 months	VTE recurrence for dabigatran vs warfarin 2.4% vs 2.1% (HR 1.10; 95% CI 0.65–1.84; p < 0.001 for non-inferiority). Major bleeding for dabigatran vs warfarin 1.6% vs 1.9% (HR 0.82; 95% CI 0.45–1.48; p = 0.38)
	Acute treatment of VTE	RE-COVER II [36]	LMWH or UFH for 5–11 days; dabigatran 150 mg bid for 6 months or LMWH or UFH for 5–11 days; warfarin (target INR 2.0–3.0) for 6 months	VTE recurrence or VTE-related death for dabigatran vs warfarin 2.3% vs 2.2% (HR 1.08; 95% CI 0.64–1.80; p < 0.001 for non-inferiority)*. Major bleeding for dabigatran vs warfarin 1.2% vs 1.7% (HR 0.69; 95% CI 0.36–1.32; p value not available)
	Secondary prevention of VTE	RE-MEDY [38]	After 3–12 months of anticoagulant therapy: Dabigatran 150 mg bid for 6–36 months or Warfarin (target INR 2.0–3.0) for 6–36 months	VTE recurrence for dabigatran vs warfarin 1.8% vs 1.3% (HR 1.44; 95% CI 0.78–2.64; p = 0.01 for non-inferiority). Major bleeding for dabigatran vs warfarin 0.9% vs 1.8% (HR 0.52; 95% CI 0.27–1.02; p = 0.06)
	Secondary prevention of VTE	RE-SONATE [38]	After 6–18 months of anticoagulant therapy: Dabigatran 150 mg bid for 6 months or Placebo for 6 months	VTE recurrence for dabigatran vs placebo 0.4% vs 5.6% (HR 0.08; 95% CI 0.02–0.25; p < 0.001). Major bleeding for dabigatran vs placebo 0.39% vs 0% (HR not available; 95% CI 0.04–1.05; p = 0.5)

*During 6 months of treatment, excluding the additional 30-day follow-up.

Abbreviations: *bid* twice daily, *CI* confidence interval, *DVT* deep vein thrombosis, *HR* hazard ratio, *INR* international normalized ratio, *LMWH* low molecular weight heparin, *NS* not significant, *od* once daily, *PE* pulmonary embolism, *RR* relative risk, *s.c.* subcutaneous, *UFH* unfractionated heparin, *VKA* vitamin K antagonist, *VTE* venous thromboembolism.

#### Rivaroxaban

Rivaroxaban is an oral, direct Factor Xa inhibitor with a rapid onset of action (maximum inhibition of Factor Xa achieved 2–4 hours post-dose) and a half-life of 5–13 hours in healthy individuals [40]. Approximately one-third of the rivaroxaban dose is excreted as unchanged drug in the urine, with the remainder of the drug metabolized and eliminated renally or by the hepatobiliary route.

Rivaroxaban has been evaluated for the initial treatment of VTE in the EINSTEIN DVT and EINSTEIN PE studies [32,33]. Both of these phase III studies were open-label, randomized, event-driven, non-inferiority studies in patients with symptomatic DVT or PE, comparing the single-drug approach with rivaroxaban (15 mg twice daily [bid] for 3 weeks then 20 mg once daily [od]) with a standard regimen of s.c. enoxaparin 1 mg/kg bid for at least 5 days overlapped with a VKA (warfarin or acenocoumarol) until the INR was at least 2.0 for 2 consecutive days. Rivaroxaban or VKA therapy was continued for 3, 6 or 12 months [32,33].

In both the EINSTEIN DVT and EINSTEIN PE studies, rivaroxaban was non-inferior to the standard enoxaparin/VKA regimen in preventing VTE recurrence (Table 2). In EINSTEIN DVT, the primary efficacy endpoint occurred in 2.1% of patients in the rivaroxaban group versus 3.0% of patients in the standard therapy group (hazard ratio [HR] 0.68; 95% confidence interval [CI] 0.44–1.04). Similarly, in EINSTEIN PE, the primary efficacy endpoint occurred in 2.1% of patients in the rivaroxaban group versus 1.8% of patients in the standard therapy group (HR 1.12; 95% CI 0.75–1.68). The incidence of the principal safety outcome of major or non-major clinically relevant bleeding was similar in patients receiving rivaroxaban compared with those receiving standard therapy in both studies (Table 2) [32,33]. The rate of major bleeding was significantly lower for rivaroxaban compared with standard therapy (1.1% vs 2.2%; p = 0.003) in EINSTEIN PE and was not significantly different in EINSTEIN DVT (0.8% vs 1.2%; p = 0.21).

A prespecified pooled analysis of the EINSTEIN DVT and EINSTEIN PE studies including more than 8000 patients permitted evaluation of clinically important subgroups and rare safety events [41]. The non-inferiority of rivaroxaban to standard therapy was confirmed (2.1% vs 2.3%; HR 0.89; 95% CI 0.66–1.19; p < 0.001), with a similar incidence of major and non-major clinically relevant bleeding (9.4% vs 10%; HR 0.93; 95% CI 0.81–1.06; p = 0.27), and importantly, a 46% relative risk reduction in major bleeding (1.0% vs 1.7%; HR 0.54; 95% CI 0.37–0.79; p = 0.002). Rivaroxaban also showed consistent efficacy and safety across key patient subgroups, irrespective of fragility, cancer or clot severity [41].

#### Apixaban

Apixaban is also an oral, direct Factor Xa inhibitor with a rapid onset and offset of action (half-life of ~12 hours after repeated dosing), with ~27% of the drug dose cleared renally [42]. The large phase III AMPLIFY trial evaluated apixaban for the initial treatment of VTE [34]. In this study, patients with symptomatic proximal DVT or PE were randomized to either monotherapy with apixaban (10 mg bid for 7 days, then 5 mg bid for 6 months) or a standard enoxaparin/warfarin regimen (s.c. enoxaparin 1 mg/kg bid until INR ≥2.0; warfarin od). Apixaban was shown to be non-inferior to standard therapy in preventing VTE recurrence or VTE-related death (2.3% vs 2.7%; relative risk [RR] 0.84; 95% CI 0.60–1.18). Apixaban was associated with a significant reduction in the principal safety outcome of major bleeding compared with standard therapy (0.6% vs 1.8%; RR 0.31; 95% CI 0.17–0.55; p < 0.001; Table 2). The composite of major and non-major clinically relevant bleeding events was also significantly lower after treatment with apixaban compared with standard therapy (4.3% vs 9.7%; RR 0.44; 95% CI 0.36–0.55; p < 0.001) [34].

#### Edoxaban

Edoxaban is another direct Factor Xa inhibitor that was evaluated in a large phase III clinical trial for use in the treatment of VTE [39]. It has a rapid onset of action, reaching maximum plasma concentration within 1–2 hours of oral administration, and has a half-life of 6–11 hours in young, healthy individuals after single doses [43]. Renal excretion accounts for 35–39% of drug metabolism. The global Hokusai-VTE study randomized patients with symptomatic DVT and/or PE to up to 12 months’ treatment with either edoxaban (60 mg od) or warfarin (target INR 2.0–3.0) after an initial treatment period with a parenteral anticoagulant (s.c. LMWH 1 mg/kg bid or 1.5 mg/kg od, or i.v. UFH, for up to 12 days) [39]. Edoxaban was shown to be non-inferior to standard therapy in preventing VTE recurrence or VTE-related death (3.2% vs 3.5%; HR 0.89; 95% CI 0.70–1.13; p < 0.001) (Table 2) and was associated with a significant reduction in the principal safety outcome of major or non-major clinically relevant bleeding (8.5% vs 10.3%; HR 0.81; 95% CI 0.71–0.94; p = 0.004 for superiority). The incidence of major bleeding was similar between edoxaban and standard therapy groups (Table 2).

Although all patients received initial treatment with a parenteral anticoagulant in the Hokusai-VTE study, the smaller phase II US multicentre study (eTris; NCT01662908) will compare 90 days’ treatment with edoxaban monotherapy (90 mg od for ~10 days, then 60 mg od for ~90 days) with a standard heparin/warfarin regimen for the treatment of symptomatic DVT. The primary endpoint in this study is magnetic resonance imaging assessment of reduction in thrombus burden at day 14–21 after commencing treatment. Recruitment is ongoing for this study.

#### Dabigatran

Dabigatran is a direct thrombin inhibitor that has been extensively evaluated in phase III studies for the treatment of VTE. Dabigatran is administered orally as the prodrug dabigatran etexilate and has a rapid onset of action with peak anticoagulant effect within 2–3 hours after administration. Dabigatran is predominantly cleared renally (~85%) and the normal half-life of 12–14 hours may be prolonged by up to twofold in patients with significant renal impairment [44].

Two large phase III trials evaluating dabigatran for the initial treatment of VTE, RE-COVER and RE-COVER II, have been completed. In both trials, patients with symptomatic DVT and PE were randomized to either dabigatran 150 mg bid or standard-intensity warfarin after an initial period of parenteral anticoagulation for a median of 9 days. Both studies demonstrated dabigatran to be non-inferior to standard therapy in preventing VTE recurrence or VTE-related death (2.4% vs 2.1%; HR 1.10; 95% CI 0.65–1.84 and 2.3% vs 2.2%; HR 1.08; 95% CI 0.64–1.80 for RE-COVER and RE-COVER II, respectively), with similar rates of major bleeding events (Table 2), but a significant reduction in major or non-major clinically relevant bleeding with dabigatran in RE-COVER (5.6% vs 8.8%; HR 0.63; 95% CI 0.47–0.84; p = 0.002) [35,36].

Efficacy and safety results from the individual RE-COVER and RE-COVER II studies were confirmed in a pooled analysis with a combined population of over 5000 patients. The incidence of recurrent VTE was 2.4% for dabigatran compared with 2.2% for warfarin-treated patients (HR 1.09; 95% CI 0.76–1.57 during 6 months of treatment). Dabigatran was also associated with a similar incidence of major bleeding (1.4% vs 2.0%; HR 0.73; 95% CI 0.48–1.11) and a lower incidence of clinically relevant bleeding (5.3% vs 8.5%; HR 0.62; 95% CI 0.50–0.76) versus warfarin. Age was shown to significantly influence the treatment effect for major or non-major clinically relevant bleeding events (p = 0.010) (see ‘Treatment of venous thromboembolism in special populations’). Treatment effects remained consistent across other patient subgroups, including in patients with PE or cancer [36].

#### Comparison of the direct oral anticoagulants with conventional therapy and each other

To date, the completed trials have reported that rivaroxaban, apixaban, edoxaban and dabigatran have similar efficacy and safety to conventional standard-of-care anticoagulation. Rivaroxaban demonstrated a significantly reduced rate of major bleeding in patients with PE, and this result was retained in the pooled analysis of EINSTEIN DVT and EINSTEIN PE data [33,41]. A significant reduction in major bleeding was also evident with apixaban in patients with DVT and/or PE [34]. Although edoxaban was associated with a significant reduction in the composite of major and non-major clinically relevant bleeding, rates of major bleeding remained similar between treatment groups [39]. Across all studies, the time spent in therapeutic range for VKA-treated patients ranged between 57–64%, a rate that compares favourably with that seen in patients in the community using standard monitoring [32-35,39]. Although patient self-monitoring and self-dose adjustment has been reported to have the potential to improve outcomes in selected patients receiving warfarin [31], this approach was not used in the comparator arms of the phase III trials with the direct oral anticoagulants.

The above data have led to both rivaroxaban and dabigatran being included in the current ACCP guidelines for the treatment of VTE, despite only rivaroxaban having been approved for this indication at the time [25]. Treatment with LMWH and a VKA is weakly recommended over rivaroxaban and dabigatran, primarily because of the lack of long-term data for these direct oral anticoagulants at the time of writing; neither rivaroxaban nor dabigatran is recommended over the other [25]. Because the direct oral anticoagulants have differing pharmacological and pharmacokinetic characteristics, patients may be more suited to one agent than another based on their own individual characteristics [45].

**Figure 1:**
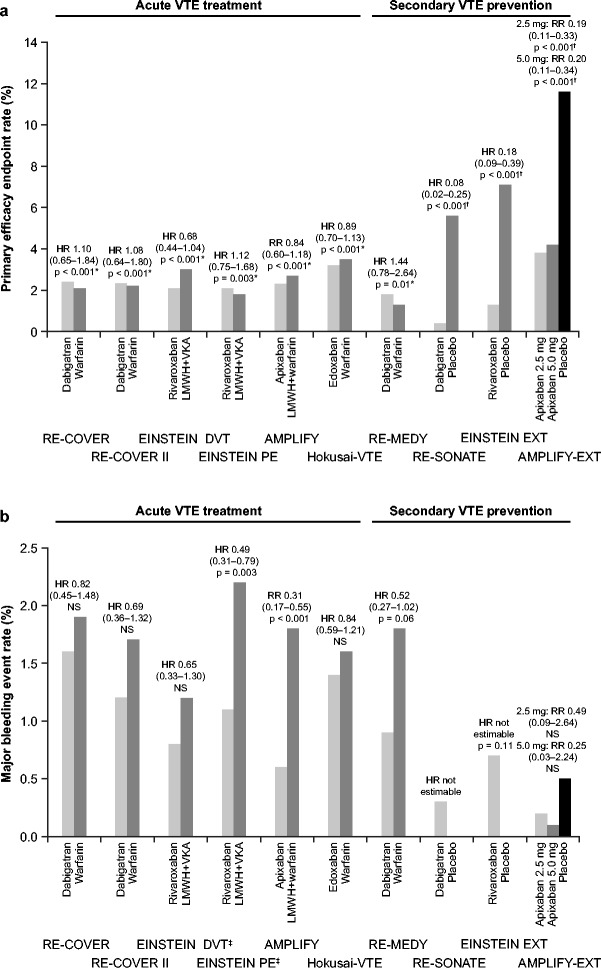
**Completed phase III trials of direct oral anticoagulants in the treatment of venous thromboembolism. (a)** Primary efficacy outcome and **(b)** Major bleeding events [32,33,35-39]. *Non-inferiority test. ^†^Superiority test. ^‡^Principal safety outcome in EINSTEIN DVT and EINSTEIN PE was major or non-major clinically relevant bleeding. HR, hazard ratio; LMWH, low molecular weight heparin; NS, not significant; RR, relative risk; VKA, vitamin K antagonist; VTE, venous thromboembolism.

The results of the phase III trials of the direct oral anticoagulants cannot be compared directly, and because head-to-head trials are unlikely, the comparative efficacy and safety of the different agents is difficult to assess (Figure 1). Although the inclusion and exclusion criteria of the different phase III trials had many similarities, these trials vary in terms of their individual study designs and patient characteristics [46]. The EINSTEIN PE and EINSTEIN DVT studies of rivaroxaban were open label, in contrast to the double-blind studies of dabigatran (RE-COVER, RE-COVER II), apixaban (AMPLIFY) and edoxaban (Hokusai-VTE) [32-35,37-39,47]. Another significant difference in the trial designs was the planned use of a single-drug approach with no initial parenteral anticoagulation in EINSTEIN DVT, EINSTEIN PE and AMPLIFY [32-34], compared with an initial treatment period with a parenteral anticoagulant (usually i.v. UFH or s.c. LMWH) in the RE-COVER and Hokusai-VTE studies [35,36,39]. In practice, because of the time required for recruitment, patients in the AMPLIFY and EINSTEIN studies were allowed to have received up to either 36 hours [34] or 48 hours [32,33] of parenteral anticoagulation prior to enrolment, respectively, with the majority receiving 24 hours of pre-study treatment. In an analysis of data from the EINSTEIN DVT and PE studies, there was no difference in outcomes between patients who received pre-randomization heparin and those who did not [48].

A large degree of variation is also seen in the number of patients with PE enrolled in the acute phase III VTE studies, and in the anatomical extent of PE at baseline. The EINSTEIN PE study recruited 4832 patients with PE (with or without DVT) [33], in comparison to the AMPLIFY [34] and the combined RE-COVER studies, which recruited 1836 and 1602 patients with PE, respectively [35,36]. In total, 3319 patients with PE (with or without DVT) were enrolled in the Hokusai-VTE study, and nearly one-third of these patients had severe PE and right ventricular dysfunction, as measured by N-terminal fragment of brain natriuretic peptide levels [39]. With regards to the anatomical extent of PE, extensive PE was defined in both the EINSTEIN PE and Hokusai-VTE studies as the involvement of multiple lobes covering at least 25% of the entire vasculature. Extensive PE was observed in 24.2% of patients in EINSTEIN PE compared with 45.8% of patients in Hokusai-VTE [33,39]. In contrast, the AMPLIFY study defined extensive PE as involving at least two lobes with at least 50% of vasculature affected for each lobe; 37.2% of patients met this criteria on presentation [34].

### Secondary prevention of venous thromboembolism

It has become clear that VTE is best considered a chronic disease, with patients being at significant risk of recurrent thrombosis after an initial event. Approximately one-third of unselected patients with VTE will experience a recurrent event within 10 years of their first episode [16,49,50]. Recurrent thrombotic events may be fatal, and non-fatal events increase the risk of developing chronic complications of VTE such as PTS and chronic thromboembolic pulmonary hypertension [7,51].

Depending on the balance between an individual’s risk of recurrent thrombosis after discontinuation of anticoagulation and their risk of bleeding while receiving treatment, continuation of anticoagulation into a “secondary prevention” phase may be justified after the initial treatment period for VTE [25]. The strongest clinical predictor of recurrence risk is the circumstance in which the initial thrombotic event occurred, in particular whether there was a temporal association with a transient provoking risk factor. In patients with proximal DVT or PE in whom a surgical or other major transient risk factor was present, the risk of recurrence is low, and 3 months’ treatment is recommended. In patients with either proximal DVT and/or PE whose initial event was unprovoked, or who have an ongoing significant persisting risk factor such as cancer, extended or long-term anticoagulation should be considered, particularly in patients with a low to moderate risk of bleeding [25]. Additional factors that would support long-term anticoagulation include male sex, the presence of PTS, significant obesity, poor underlying cardiovascular reserve and a history of more than one prior thrombotic event [52,53].

#### Direct oral anticoagulants for the secondary prevention of venous thromboembolism

The level of adherence to treatment guidelines for long-term management of VTE in the outpatient setting is unclear [54]. Factors that might limit the acceptance of extended anticoagulation include the current requirement for therapeutic monitoring and dietary restrictions with warfarin, along with patients’ and clinicians’ perception of the bleeding risk associated with anticoagulation. Possibly, more convenient anticoagulant agents with at least equivalent efficacy and safety may lead to more widespread acceptance of extended anticoagulation. A number of studies have evaluated the direct oral anticoagulants for the secondary prevention of VTE [32,37,38].

The EINSTEIN EXT study evaluated rivaroxaban for secondary prevention after initial anticoagulant treatment for 6–12 months, and found that treatment with rivaroxaban 20 mg od for an additional 6 or 12 months was associated with a significant 82% reduction in the relative risk of VTE recurrence compared with placebo, without a significantly increased risk of major bleeding (Table 2) [32]. A net clinical benefit, defined as the composite of the primary efficacy outcome or major bleeding, was observed with rivaroxaban compared with placebo (2.0% vs 7.1%, p < 0.001).

The placebo-controlled AMPLIFY-EXT study assessed apixaban (2.5 mg bid or 5 mg bid for 12 months) for secondary prevention of VTE after 6–12 months’ initial treatment [37]. Symptomatic recurrent VTE or VTE-related death occurred in significantly fewer patients receiving apixaban than those given placebo: 73/829 patients (8.8%) in the placebo arm, 14/840 patients (1.7%) in the 2.5 mg apixaban arm and 14/813 patients (1.7%) in the 5 mg apixaban arm (p < 0.001 for both apixaban doses vs placebo). The rates of major bleeding were similar for apixaban compared with placebo (2.5 mg group vs placebo: 0.2% vs 0.5%; RR 0.49; 95% CI 0.09–2.64; 5 mg group vs placebo: 0.1% vs 0.5%; RR 0.25; 95% CI 0.03–2.24; p-values not reported). For the first time in a study of secondary prevention of VTE, anticoagulant therapy was demonstrated to reduce the risk of all-cause mortality [37]. The lower apixaban dose of 2.5 mg bid, the same dose that was used in orthopaedic prophylaxis studies, appeared to have similar efficacy to the higher apixaban dose in preventing recurrent thrombosis, and the risk of both major and non-major clinically relevant bleeding did not differ significantly from that observed in patients receiving placebo.

Extended-duration dabigatran treatment has also been evaluated for the secondary prevention of VTE compared with both warfarin (RE-MEDY) and placebo (RE-SONATE) [38]. RE-MEDY demonstrated that dabigatran 150 mg bid was non-inferior to warfarin in preventing VTE recurrence after 3–12 months’ prior anticoagulant therapy [38]. There was a significant reduction in major or non-major clinically relevant bleeding events and a non-significant reduction in major bleeding. A higher incidence of acute coronary syndrome events was reported in patients receiving dabigatran in this study (0.9% vs 0.2% for warfarin; p = 0.02). A subsequent meta-analysis of nine randomized controlled trials of dabigatran for a number of indications demonstrated a modest increase in the risk of myocardial infarction and acute coronary syndrome with dabigatran compared with warfarin (odds ratio (OR) 1.30; 95% CI 1.04–1.63; p = 0.021) but not with apixaban or rivaroxaban [55]. The RE-SONATE study showed that after 6–18 months’ initial anticoagulant therapy, an 82% reduction in recurrent or fatal VTE in patients receiving dabigatran 150 mg bid occurred without an increase in the risk of major bleeding [38].

All three direct oral anticoagulants evaluated for the secondary prevention of recurrent VTE appear to be effective agents for this indication, with an acceptable risk of bleeding (Figure 1). The choice of agent is likely to be influenced by availability and individual patient characteristics as discussed below.

### Treatment of venous thromboembolism in special populations

#### Elderly patients

Because the risk of VTE increases exponentially with age, there is an increasing need for effective treatment in elderly patients [2,9,16]. In addition, treatment of older patients is often complicated by co-morbidities, concomitant medication and impaired renal function. The pharmacokinetics of the direct oral anticoagulants are altered in the healthy elderly population compared with in younger individuals. The half-life of rivaroxaban is prolonged in elderly versus younger individuals (11–13 hours vs 5–9 hours), and there is an approximately twofold increase in the area under the concentration–time curve of dabigatran and a one-third increase in that of apixaban in older individuals [40,42,44]. There is no routine requirement for dose adjustment based on age with rivaroxaban provided renal function is adequate [40], and the EINSTEIN clinical trial programme did not exclude elderly patients (age >75 years). In total, the EINSTEIN DVT and EINSTEIN PE studies included >3000 patients aged >65 years, ~1300 of whom were aged >75 years [32,33]. Subgroup analyses from the EINSTEIN DVT and EINSTEIN PE pooled data showed that fragile patients (those aged >75 years or with CrCl <50 ml/min or body weight ≤50 kg) had significantly reduced rates of major bleeding with rivaroxaban versus enoxaparin/VKA (1.3% vs 4.5%; HR 0.27; 95% CI 0.13–0.54; p = 0.011); this result was consistent across the individual components of this subgroup. The net clinical benefit for patients treated with rivaroxaban was also higher versus enoxaparin/VKA in fragile patients (4.6% vs 8.4%; HR 0.51; 95% CI 0.34–0.77; p = 0.017) [41].

Supplementary information from the RE-COVER, RE-COVER II, RE-MEDY and RE-SONATE trials states that 2892 patients aged ≥65 years, including at least 788 patients ≥75 years, were enrolled in these trials [36,38]. Age was shown to have no effect on recurrent VTE or bleeding outcomes [36,56].

The AMPLIFY-EXT study enrolled 819 patients aged >65 years, of whom 329 were >75 years [37]. Subgroup analysis suggested similar efficacy of both the apixaban 2.5 mg and 5.0 mg doses, with a trend towards increased rates of major and non-major clinically relevant bleeding in patients >75 years of age with both doses compared with placebo. In Hokusai-VTE, a total of 1104 patients aged >75 years were enrolled. In this subgroup, efficacy and safety outcomes were similar between patients receiving edoxaban 60 mg od or warfarin [39].

#### Patients with renal impairment

All the direct oral anticoagulants are excreted renally to some extent; therefore, reduced renal function can result in excessive accumulation of these agents [57,58], with an associated increased risk of bleeding.

All published studies to date examining the direct oral anticoagulants for either the initial treatment or secondary prevention of VTE have excluded patients with severe renal impairment, defined as a calculated CrCl of <30 ml/min in the rivaroxaban, edoxaban and dabigatran studies, and of <25 ml/min (or a serum creatinine level of >2.5 mg/dl) in the trials examining apixaban [32,33,35-37]. Between ~5% and 10% of patients in the studies of direct oral anticoagulants (EINSTEIN studies of rivaroxaban, AMPLIFY/AMPLIFY-EXT studies of apixaban, Hokusai-VTE study of edoxaban and RE-COVER studies of dabigatran) had moderate renal impairment (CrCl 30–50 ml/min). Published supplementary data for the initial VTE treatment studies reported no statistically significant differences in efficacy and safety of the direct oral anticoagulants administered at standard doses in this patient group compared with those with normal renal function; however, the number of patients was small.

Assessment of renal function is recommended before initiating treatment with any of the direct oral anticoagulants, and in patients on long-term treatment when conditions arise that may affect renal function [58]. Although a lower dose of rivaroxaban (15 mg od) has been evaluated for the prevention of stroke in patients with atrial fibrillation (AF), and is recommended for patients with moderate (CrCl 30–49 ml/min) or severe (CrCl 15–29 ml/min) renal impairment [40,59], this has not been studied in a clinical setting for the treatment of VTE. The European Medicines Agency (EMA), therefore, only recommends a dose reduction to 15 mg od for the secondary prevention of VTE in patients with moderate to severe renal impairment if the risk of bleeding outweighs the risk of recurrent VTE (based on pharmacokinetic modelling data) [40]. Because of the lack of efficacy data, the US Food and Drug Administration (FDA) suggests avoiding the use of rivaroxaban in patients with CrCl <30 ml/min, and does not recommend dose adjustment based on renal function in the VTE treatment indication [59]. Dabigatran is contraindicated in Europe for patients with severe renal impairment, and a dose reduction to 75 mg bid is recommended by the FDA for stroke prevention in patients with non-valvular AF and CrCl 15–30 ml/min (based on pharmacokinetic modelling data). Dose reductions are also recommended by the EMA for patients with moderate renal impairment. [44,60]. For apixaban, use in its current indications is not recommended in patients with a CrCl <15 ml/min, and dose reduction is only recommended in patients with non-valvular AF with at least two of the following characteristics: age ≥80 years, body weight ≤60 kg or serum creatinine ≥1.5 mg/dl [61]. Reduced dosing was not part of the study design in the RE-COVER and AMPLIFY trials [34,35], but in Hokusai-VTE edoxaban given at a halved dose of 30 mg od for patients with CrCl 30–50 ml/min was associated with similar efficacy to standard of care, together with a significant reduction in major and non-major clinically relevant bleeding [39].

#### Pregnant patients

Although not yet evaluated extensively in pregnancy, based on limited data and their small molecular weight [40,58], it is likely that all the direct oral anticoagulants cross the placenta and, therefore, have the potential for teratogenicity and unwanted foetal anticoagulant effects. Therefore, LMWH or UFH remain the agents of choice for VTE during pregnancy or breastfeeding, because neither of these agents crosses the placental barrier or is secreted in breast milk [26]. Warfarin is contraindicated during pregnancy owing to its teratogenic effects, particularly during weeks 6–13 of gestation, but can be taken while breastfeeding [26].

#### Patients with active malignancy

Active malignancy is a significant risk factor for thrombosis and is present in ~20% of cases of VTE. The development of VTE is known to increase mortality rates and treatment costs for patients with cancer [17]. Extended treatment with LMWH for at least 6 months is the treatment of choice for patients with malignancy-associated VTE, owing to the finding of a ≤50% reduction in the risk of recurrent thrombosis with this approach compared with warfarin. In published trials examining the direct oral anticoagulants for the treatment of VTE, ~5% of patients had active malignancy at the time of enrolment; therefore, the experience to date with the direct oral anticoagulants in patients with cancer is limited. In all these studies, patients in the comparator arm received warfarin rather than LMWH for extended treatment. [25]. The largest dataset of patients with cancer from studies of direct oral anticoagulants for the treatment of VTE comes from a pooled analysis of the EINSTEIN DVT and EINSTEIN PE studies. A total of 655 patients with active cancer (at baseline or diagnosed during the study) and 469 patients with a history of cancer were randomized. Patients received either rivaroxaban 15 mg bid for 21 days then 20 mg od, or enoxaparin 1 mg/kg bid overlapping and transitioning to warfarin or acenocoumarol (INR 2.0–3.0) for 3, 6 or 12 months. In patients with active cancer (diagnosed at baseline or during treatment), the incidence of recurrent venous thromboembolic and clinically relevant bleeding events were numerically lower in patients allocated to rivaroxaban compared with patients assigned to enoxaparin/VKA (5% vs 7%; HR 0.67; 95% CI 0.35–1.30 and 14% vs 16%; HR 0.80; 95% CI 0.54–1.20, respectively). Patients with active cancer receiving rivaroxaban had a significantly lower incidence of major bleeding than those receiving standard therapy (2% vs 5%; HR 0.42; 95% CI 0.18–0.99), whereas the incidence of all-cause death was similar between the rivaroxaban and standard therapy groups (16% vs 18%; HR 0.93; 95% CI 0.64–1.35). Overall, rivaroxaban had a significant advantage compared with enoxaparin/VKA in patients with active cancer with regard to major bleeding and net clinical benefit, consistent with the overall result of the pooled analysis of the EINSTEIN DVT and EINSTEIN PE studies [41,62]. Using data from AMPLIFY, a subgroup analysis was performed to compare the efficacy and safety of apixaban (10 mg bid for 7 days followed by 5 mg bid) with conventional treatment (enoxaparin 1 mg/kg bid for at least 5 days followed by dose-adjusted warfarin [target INR 2.0–3.0]) in patients with and without active cancer at baseline. In patients with active cancer at baseline (3.1% of the total randomized population; N = 5395), recurrent VTE occurred in 3/81 (3.7%) patients in the apixaban group and in 5/78 (6.4%) patients in the conventional treatment group (RR 0.56; 95% CI 0.13–2.37); major bleeding occurred in 2/87 (2.3%) and 4/80 (5.0%) patients, respectively (RR 0.45; 95% CI 0.08–2.46). Although the number of cancer patients was small, the results of this prespecified subgroup analysis suggest that apixaban is as effective as conventional therapy utilizing warfarin in patients with both VTE and active cancer, and is associated with fewer bleeding events [63]. It should be emphasized that there are no current data directly comparing the direct oral anticoagulants with continued therapy with LMWH in patients with malignancy-associated thrombosis.

#### Low or high body weight

Although specific data for the use of direct oral anticoagulants in patients with very low or very high body weight are lacking, no exclusion criteria for body weight have been specified in published phase III trials [32,33,35]. A subgroup analysis of the EINSTEIN DVT study found that rivaroxaban had consistent efficacy and risk of bleeding across body weight categories [32]. Supplementary data from the apixaban studies reported similar findings [34,37], as did pooled results from the RE-COVER trials for the primary efficacy outcome (subgroup safety outcomes were not reported) [36]. All studies enrolled limited numbers of patients with low body weight, defined as either <50 kg or ≤60 kg. For this reason, and because of the potential to overestimate renal function in patients with low body weight when using standard measurements such as glomerular filtration rate [64], care should be taken in administering the direct oral anticoagulants to patients with very low body weight, despite dose adjustment not being routinely recommended in the product information [40,42,44].

#### The impact of concomitant medications

Although the direct oral anticoagulants have considerably fewer drug interactions than VKAs, certain drugs may interfere with their metabolism and elimination. Apixaban, rivaroxaban and dabigatran are substrates of P-glycoprotein, and apixaban and rivaroxaban are also substrates of cytochrome P450 3A4. Inhibitors or inducers of these enzymes have the potential to interact with these anticoagulants; therefore, caution is required when treating patients with such medications [40,42,44].

Administration of other anticoagulant drugs should be avoided with all the direct oral anticoagulants. Concurrent use of acetylsalicylic acid (ASA) has been shown to increase the risk of haemorrhage in patients with AF treated with the direct oral anticoagulants. Concomitant use of ASA should be restricted to carefully selected patients in whom there is a clear indication for ongoing antiplatelet therapy. As with warfarin, concurrent use of the direct oral anticoagulant agents with both ASA and a P2Y_12_ inhibitor is associated with a significant increase in bleeding risk; therefore, triple therapy should be avoided where possible or given for the minimum duration possible [65]. A post hoc analysis assessed the impact of combined anticoagulant and ASA or non-steroidal anti-inflammatory drug (NSAID) therapy on bleeding risk compared with anticoagulant therapy alone in patients enrolled in EINSTEIN DVT and EINSTEIN PE. The safety population of the EINSTEIN DVT and PE studies comprised 8246 patients (4130 assigned to rivaroxaban and 4116 assigned to enoxaparin/VKA); of these patients, 1884 (22.8%) and 1202 (14.6%) received concomitant ASA or NSAID therapy at any time during their study treatment, respectively. During NSAID/anticoagulant concomitant treatment, clinically relevant and major bleeding occurred with an event rate of 37.5 per 100 patient-years versus 16.6 per 100 patient-years during anticoagulant use only (HR 1.77; 95% CI 1.46–2.14) and 6.5 per 100 patient-years versus 2.0 per 100 patient-years during anticoagulant use only (HR, 2.37; 95% CI 1.51–3.75), respectively. Similarly, during ASA/anticoagulant concomitant treatment, clinically relevant and major bleeding occurred with an event rate of 36.6 per 100 patient-years versus 16.9 per 100 patient-years during anticoagulant use only (HR 1.70; 95% CI 1.38–2.11) and 4.8 per 100 patient-years versus 2.2 per 100 patient-years during anticoagulant use only (HR 1.50; 95% CI 0.86–2.62), respectively. Increases in the risk of clinically relevant and major bleeding were similar for rivaroxaban and enoxaparin/VKA treatment regimens. These data indicate that physicians should inform patients about the potential for increased bleeding with concomitant use of NSAIDs or ASA and advise them on their treatment accordingly [66].

### Practical considerations: laboratory testing and bleeding management

When considering the practical differences between direct oral anticoagulant and VKA therapy, all four direct oral agents have the advantage of not requiring routine coagulation monitoring along with fixed-dose regimens. Therefore, these agents offer simplified, more convenient therapy for VTE compared with conventional standard of care. Poor INR control with VKA therapy can result in sub-therapeutic anticoagulation, increasing the risk of recurrent VTE or bleeding complications. It has also been demonstrated that sub-optimal anticoagulation with warfarin therapy may be associated with a risk of PTS in patients with DVT [67]. The results of small studies suggest that continued treatment with LMWH is associated with a reduction in the risk of PTS, possibly as a result of improved consistency of anticoagulant effect during the initial treatment period [68]. It is theoretically possible that the potential for reduced variability in anticoagulant effect with the direct oral anticoagulants may result in a similar benefit; however, to date, the incidence of PTS has not been reported in trials of the direct oral anticoagulants and further studies are required to test this hypothesis.

While the lack of a need for routine coagulation monitoring is an advantage for the majority of patients, the absence of a regular measure of drug effect or plasma levels means that patient compliance is difficult to assess. Patients who have a history or tendency of non-compliance may be better suited to VKA therapy with regular INR monitoring to reinforce compliance, although this may require additional resources such as domiciliary nurse visits to measure INR. INR is not a valid measure to assess the anticoagulant activity of the direct OACs; however, it should be noted that tests are available for both qualitative and/or quantitative measurement of direct oral anticoagulants if assessment of drug exposure is required in certain clinical situations or patient populations [69].

The activated partial thromboplastin time may provide a qualitative assessment of dabigatran plasma levels [70], although limitations include high interindividual variability and variability due to testing equipment used [71-73]. Alternatively, quantitative assessment can be obtained through the HEMOCLOT® assay: a diluted thrombin time test with appropriate calibrators for the measurement of dabigatran plasma levels [70].

For rivaroxaban, the prothrombin time may provide a qualitative assessment of drug exposure when rivaroxaban-sensitive reagents are used, although the sensitivity of the assay varies between reagents. A quantitative measure of rivaroxaban levels can be obtained using an anti-Factor Xa chromogenic assay with commercially available calibrators and controls. Both low and high plasma levels can be measured with acceptable inter-laboratory precision using this quantitative approach [74]. Anti-Factor Xa chromogenic assays with specific calibrators can also provide quantitative measurements of apixaban plasma levels [75]. For all direct oral anticoagulants, it is important to note that the interval between the last dose and blood sampling time, in addition to renal function status, must be considered when interpreting the test results.

A concern of many physicians regarding the use of anticoagulation is the current lack of a reversal agent and the impact that this may have in situations such as life-threatening bleeding events or in patients who require urgent surgery. VKAs represent the leading cause of hospital admission for adverse events [76], and hospitalization due to intracranial haemorrhage (ICH) in patients treated with VKAs is associated with a high in-hospital mortality rate [77,78]. Currently, supportive care measures such as adequate hydration, appropriate transfusion support, and if possible the identification and control of the site of bleeding, are recommended as the mainstay of management for bleeding occurring in patients receiving direct oral anticoagulants [79]. It should be noted that because of the relatively short half-life of these agents, there is minimal anticoagulant effect 24 hours after an initial dose or 12 hours after bid dosing, provided renal impairment is not present. The clinical evidence for the use of pro-haemostatic agents, such as activated or non-activated prothrombin complex concentrates or recombinant Factor VIIa, is still limited to a small number of case reports and preclinical data or studies in healthy subjects, and these products should only be given consideration in cases of life-threatening bleeding [79]. A phase II study with a recombinant Factor Xa protein (PRT064445, andexanet alfa) as a specific reversal agent for rivaroxaban is ongoing. Interim results have been reported [80], and enrolment has begun in a phase III study of its safety and efficacy [81]. Phase III studies with rivaroxaban (ANNEXA-R) and apixaban (ANNEXA-A) are also underway. RE-VERSE AD (RE-VERSal Effects of Idarucizumab on Active Dabigatran) is a phase III, interventional, open-label trial to evaluate the reversal of the anticoagulant effects of dabigatran. In this trial, 5.0 g i.v. idarucizumab is administered to patients treated with dabigatran etexilate who have uncontrolled bleeding or require emergency surgery or procedures (NCT 02104947).

The incidence of critical site bleeding events represents an important measure in clinical studies on anticoagulants, as the risk of mortality after a bleeding event is closely related to the site of haemorrhage. For example, non-access-site bleeding events may have a more significant impact on the likelihood of death than access-site bleeding events [82]. ICH is a rare yet devastating adverse event related to anticoagulation treatment that can lead to severe disability and mortality. In an analysis of the 30-day survival rate of patients with AF on and off anticoagulation, warfarin therapy was associated with an increased mortality from ICH compared with patients receiving no anticoagulation (OR 1.62; 95% CI 0.88–2.98), despite the availability of vitamin K and fresh frozen plasma for the reversal of VKA activity [83]. In studies examining rivaroxaban, apixaban and dabigatran for the prevention of stroke in patients with AF, all three agents demonstrated a reduced risk of ICH compared with warfarin. In the EINSTEIN PE, AMPLIFY, Hokusai-VTE and RE-COVER trials, which reported on ICH as a separate endpoint, the incidence was lower with the direct oral anticoagulants than with warfarin (3/2412 [0.1%] vs 12/2405 [0.5%] patients in EINSTEIN PE; 3/2676 [0.1%] vs 6/2689 [0.2%] in AMPLIFY; 5/4118 [0.1%] vs 18/4122 [0.4%] in Hokusai-VTE; 0/1274 [0.0%] vs 3/1265 [0.2%] in RE-COVER), although this was not assessed statistically [33-36,39]. Ongoing post-marketing surveillance will be needed to clarify if this potential advantage is confirmed in patients receiving treatment for VTE and to further examine the case–fatality rate of major bleeding in patients receiving a direct oral anticoagulant.

Real-world studies allow the risk of bleeding to be assessed in routine clinical practice as opposed to the controlled conditions of clinical studies. For example, the prospective Dresden NOAC Registry, which enrols patients indicated for at least 3 months’ anticoagulation therapy with a direct oral anticoagulant, has recently published results on the rates, management and outcome of bleeding complications during rivaroxaban therapy in daily care. Of the 2346 patients enrolled between October 2011 and December 2013, 575 were prescribed rivaroxaban for VTE treatment, and the rate of major bleeding for these patients was 4.1%/year (95% CI 2.5–6.4) [84]. This incidence was higher than the rate of major bleeding reported in the EINSTEIN studies (1.0% in total) [41]; however, patients in the Dresden NOAC Registry were older and may have received longer durations of anticoagulant treatment and follow-up. Of 1082 bleeding events across 762 patients with AF and VTE, approximately 60% were managed conservatively. Although approximately 40% of major bleeding events required surgical or interventional treatment, procoagulant therapy was rarely needed [84]. There is scant real-life evidence data for the direct oral anticoagulants dabigatran, apixaban and edoxaban. A recent retrospective cohort study compared the risk of gastrointestinal (GI) bleeding in 374 patients, of whom 147 received rivaroxaban and 227 dabigatran in a community hospital setting. This first head-to-head study showed that GI bleeding occurred in 5.3% of patients in the dabigatran group compared with 4.8% of patients in the rivaroxaban group (p = 0.8215). Both drugs had a higher bleeding risk in the first 40 days. In particular, multivariate analysis showed that the odds of experiencing a GI bleeding event while on dabigatran for ≤40 days when compared to ≥40 days was 8.3 (p < 0.0001). In the rivaroxaban group, patients who were on the drug for ≤40 days had a higher incidence of bleeding compared with those taking the drug for >40 days (OR 2.8; p = 0.023) [85].

## Conclusions

Anticoagulant therapy remains the central component of the management of patients with VTE. Available data confirm that the direct oral anticoagulants appear to offer a realistic alternative to the traditional management strategy of a parenteral anticoagulant followed by a VKA. Further information from ongoing clinical trials in patients treated with these agents will help clarify the role of these drugs. As therapeutic choices widen, clinical judgement regarding the optimal choice of anticoagulant based on individual patient characteristics will become increasingly important.

## Abbreviations

ACCP: American College of Chest Physicians
AF: Atrial fibrillation
bid: Twice daily
CI: Confidence interval
CrCl: Creatinine clearance
DVT: Deep vein thrombosis
EMA: European Medicines Agency
FDA: US Food and Drug Administration
HR: Hazard ratio
ICH: Intracranial haemorrhage
INR: International normalized ratio
i.v.: Intravenous
LMWH: Low molecular weight heparin
NR: Not reported
NS: Not significant
od: Once daily
OR: Odds ratio
PE: Pulmonary embolism
PTS: Post-thrombotic syndrome
RR: Relative risk
s.c.: Subcutaneous
UFH: Unfractionated heparin
VKA: Vitamin K antagonist
VTE: Venous thromboembolism
